# Pulse-pressure variation and hemodynamic response in patients with elevated pulmonary artery pressure: a clinical study

**DOI:** 10.1186/cc9060

**Published:** 2010-06-11

**Authors:** Moritz Wyler von Ballmoos, Jukka Takala, Margareta Roeck, Francesca Porta, David Tueller, Christoph C Ganter, Ralph Schröder, Hendrik Bracht, Bertram Baenziger, Stephan M Jakob

**Affiliations:** 1 Department of Intensive Care Medicine, Bern University Hospital and University of Bern (Inselspital), Freiburgstrasse 10, 3010 Bern, Switzerland; 2 Department of Anesthesiology and Pain Therapy, Bern University Hospital and University of Bern (Inselspital), Freiburgstrasse 10, 3010 Bern, Switzerland

## Abstract

**Introduction:**

Pulse-pressure variation (PPV) due to increased right ventricular afterload and dysfunction may misleadingly suggest volume responsiveness. We aimed to assess prediction of volume responsiveness with PPV in patients with increased pulmonary artery pressure.

**Methods:**

Fifteen cardiac surgery patients with a history of increased pulmonary artery pressure (mean pressure, 27 ± 5 mm Hg (mean ± SD) before fluid challenges) and seven septic shock patients (mean pulmonary artery pressure, 33 ± 10 mm Hg) were challenged with 200 ml hydroxyethyl starch boli ordered on clinical indication. PPV, right ventricular ejection fraction (EF) and end-diastolic volume (EDV), stroke volume (SV), and intravascular pressures were measured before and after volume challenges.

**Results:**

Of 69 fluid challenges, 19 (28%) increased SV > 10%. PPV did not predict volume responsiveness (area under the receiver operating characteristic curve, 0.555; *P *= 0.485). PPV was ≥13% before 46 (67%) fluid challenges, and SV increased in 13 (28%). Right ventricular EF decreased in none of the fluid challenges, resulting in increased SV, and in 44% of those in which SV did not increase (*P *= 0.0003). EDV increased in 28% of fluid challenges, resulting in increased SV, and in 44% of those in which SV did not increase (*P *= 0.272).

**Conclusions:**

Both early after cardiac surgery and in septic shock, patients with increased pulmonary artery pressure respond poorly to fluid administration. Under these conditions, PPV cannot be used to predict fluid responsiveness. The frequent reduction in right ventricular EF when SV did not increase suggests that right ventricular dysfunction contributed to the poor response to fluids.

## Introduction

A main goal of cardiovascular support is to restore and maintain sufficient cardiac output. Whereas prompt treatment of hypovolemia is necessary to sustain tissue perfusion, too much volume administration may result in edema formation and impaired tissue perfusion, with consequent organ dysfunction and increased risk of death [1-5]. Because blood volume cannot reliably be assessed clinically [6-11], the hemodynamic response to a volume challenge is commonly used to guide fluid management. Pulse-pressure variation (PPV), reflecting variations in stroke volume, has been advocated to discriminate between patients whose stroke volume increases in response to volume expansion and those who do not respond [12]. Conversely, PPV due to increased right ventricular afterload and right ventricular dysfunction may misleadingly suggest volume responsiveness [13-15]. Increased pulmonary artery pressure and moderate or transient right ventricular dysfunction are not rare in intensive care patients requiring hemodynamic support. For example, transient right ventricular dysfunction occurs after cardiac surgery [16,17], and high pulmonary artery pressures and dysfunction of both ventricles are common in septic shock [18,19].

We hypothesized that PPV does not predict volume responsiveness in the presence of increased pulmonary artery pressure, and that right ventricular dysfunction may contribute to this. To test this hypothesis, we measured PPV and the hemodynamic response to clinically indicated fluid challenges in patients with septic shock and in postoperative cardiac surgery patients with increased pulmonary artery pressure.

## Materials and methods

The study was approved by the Ethics Committee of the Canton of Bern. Written informed consent was obtained from 20 cardiac surgery patients with a preoperative history of increased pulmonary artery pressure, and from both an independent physician and a close family member of 10 patients in septic shock.

### Inclusion criteria

Cardiac surgery patients were included if they had an increased pulmonary artery pressure, as estimated during a preoperative echocardiogram (peak pulmonary artery pressure, ≥40 mm Hg) or a history of right ventricular myocardial infarction or right heart failure, and a clinical indication for a pulmonary artery catheter perioperatively, as judged by the patient's cardiac anesthetist, who was not involved in the study.

Patients with septic shock had septic shock defined as three of four systemic inflammatory-response syndrome criteria: (a) hyperthermia (≥38°C) or hypothermia (≤5.6°C); (b) tachycardia (heart rate, ≥90 beats/min); (c) tachypnea (respiratory rate, ≥20 breaths/min) or need for mechanical ventilation; and (d) leukocytosis (white blood cell count, ≥10 × 10^3 ^per microliter) or leucopenia (white blood cell count, ≤3 × 10^3 ^per microliter), and mean blood pressure < 60 mm Hg, despite volume resuscitation, or need for vasopressors to keep mean blood pressure ≥60 mm Hg. In addition, the patient had to have a clinically indicated pulmonary artery catheter ordered by the clinical team treating the patient.

All patients were receiving controlled mechanical ventilation with a tidal volume of 8-10 ml/kg and a ventilatory frequency that resulted in a normal arterial pCO_2_.

### Exclusion criteria

Patients with severe mitral valve insufficiency were excluded.

### Measurement of cardiac output, stroke volume, right-ventricular end-diastolic volume and ejection fraction, and blood pressures

A volumetric pulmonary artery catheter (Edwards Combo V Catheter, Edwards Lifesciences LLC, Irvine, CA, USA) and cardiac output monitor (Vigilance, Edwards Lifesciences) were used to measure cardiac output, pulmonary artery pressures, and right ventricular ejection fraction and end-diastolic volume. Systemic and pulmonary artery and central venous pressures were recorded with quartz pressure transducers and displayed continuously on a multimodular monitor (Merlin; Hewlett Packard, Geneva, Switzerland). The pressure transducers were zeroed to the level of the heart. Heart rate was measured from the electrocardiogram (ECG), which was continuously monitored. Only the variables routinely available by using the patient-monitoring devices were made available to and were used by the clinical team.

For the study purposes, the intravascular pressures and heart rate were also recorded at 50 Hz on a computer using AcqKnowledge software (version 3.8.1; Biopac Systems, Goleta, CA, USA), and variables from the continuous cardiac-output monitor/mixed venous oxygen-saturation monitor (Vigilance; Edwards Lifesciences) were recorded with a frequency of 0.5 Hz on the same computer by using the serial output from the monitor and a proprietary research software (Edwards Lifesciences) and analyzed off-line.

### Study protocol

If the intensivist in charge of the care of the patient considered a volume challenge to be clinically indicated, 200 ml of hydroxyethyl starch (Voluven 6%, Fresenius Kabi AG, Stans, Switzerland) were infused over a 10-minute period. The study personnel performed hemodynamic measurements immediately before and between 15 and 20 minutes after the end of the volume challenge. The ventilator airway-pressure signal on the ventilator screen and the end-tidal CO_2 _signal displayed on the monitor were visually observed, and fluid challenges were included only if signs of spontaneous respiratory efforts were absent.

The semicontinuous cardiac-output values were obtained from the values captured from the serial port of the device (equivalent to STAT mode). For each variable, artifact-free 2-minute mean values were used for analysis. The main reason(s) that the clinician in charge of the patient considered the fluid challenge necessary was recorded.

### Assessment of pulse-pressure variation

PPV analysis was performed on the arterial pressure curve of 20 consecutive heart beats by using the following algorithm (20): PPV (%) = 100 × (Pp_max _- Pp_min_)/((Pp_max _+ Pp_min_)/2). Only recordings made during volume-controlled mechanical ventilation were analyzed. Each beat-to-beat tracing of intravascular central venous and pulmonary artery pressures was independently assessed by two senior investigators (SJ and JT, blinded for the response to volume challenge) to verify the absence of any spontaneous respiratory activity.

### Evaluation of volume response

Changes in stroke volume (SV) were used to define response to volume challenge. An increase in stroke volume exceeding 10% after the volume challenge was considered a positive response. The volume challenge should increase the SV as a result of acutely increased preload in a heart operating on the steep portion of the cardiac-function curve [20,21]. The results were therefore analyzed in two ways: including all volume challenges, and including only those resulting in an increase in central venous pressure (CVP) of > 1 mm Hg. The directional changes in right ventricular ejection fraction and end-diastolic volume in response to volume challenge were separately analyzed for the responders and nonresponders.

### Statistics

To assess differences in proportions between groups of patients, Fisher's Exact test was used. The effect of a volume challenge on hemodynamic variables was assessed with ANOVA for repeated measurements by using one within-subject factor (hemodynamic variable) and two between-subject factors (responder versus nonresponder with respect to stroke volume, and diagnosis). With this approach, a different response of a given hemodynamic variable in responders versus nonresponders is indicated by a fluid challenge-responder interaction, and a difference in the behavior of responders versus nonresponders in the two patient groups is indicated by a fluid challenge/responder/diagnosis interaction. Receiver operating characteristic (ROC) curves were constructed to evaluate the predictive value of PPV. Best predictive threshold was defined as the highest sum of sensitivity and specificity. Data are presented as median and interquartile range (demographics and ventilator settings), as percentage (proportional data), and as mean ± SD (hemodynamic variables). A *P *value < 0.05 was considered statistically significant.

## Results

Four cardiac surgery patients and two septic shock patients were excluded from the data analysis because of cardiac dysrhythmia during the study. Further, one cardiac surgery and one septic shock patient were excluded because of continuous spontaneous respiratory activity. Accordingly, 15 postoperative cardiac surgery patients and seven patients with septic shock were analyzed. Patient data are indicated in Table 1.

**Table 1:** Patient characteristics and ventilator settings during the study

	Cardiac surgery (**n **= 15)	Septic shock (**n **= 7)
Age (years)	76 (68 to 79)	72 (61 to 73)
SAPS II	31 (24 to 35)	74 (49 to 100)
SOFA		14 (13 to 15)
Tidal volume (ml/kg)	9 (8 to 10)	9 (8 to 10)
PEEP (cm H_2_O)	5 (5 to 5)	7.5 (7.5 to 10)
Inspiratory plateau pressure (cm H_2_O)	21 (17 to 26)	24 (18 to 37)
Main diagnosis (cardiac surgery) and source of sepsis (*n*)	Aortic valve stenosis (9) Coronary artery disease (5) Mitral valve insufficiency (1)	Abdominal (4) Lung (1) Unknown (2)

Data are presented as median (interquartile range).

PEEP, positive end-expiratory pressure; SAPS, simplified acute physiology score; SOFA, sequential organ-failure assessment.

Sixty-nine fluid challenges were performed (44 in cardiac surgery patients and 25 in septic shock patients; one to eight per patient). The most common indications for fluid challenge were hypotension and peripheral vasoconstriction (Additional file 1).

Baseline hemodynamics (before the first fluid challenge) were similar in cardiac surgery and septic patients (Table 2). Overall, the fluid challenges increased stroke volume, CVP, pulmonary artery occlusion pressure (PAOP), and systemic and pulmonary arterial pressures (Table 3).

**Table 2:** Baseline hemodynamic characteristics

	Cardiac surgery	Sepsis
Stroke volume (ml/m^2^)	28 ± 9	33 ± 5
Pulmonary artery occlusion pressure (mm Hg)	14 ± 4	15 ± 5
Central venous pressure (mm Hg)	12 ± 5	13 ± 6
Mean pulmonary arterial pressure (mm Hg)	27 ± 5	33 ± 10
Mean arterial pressure (mm Hg)	70 ± 10	62 ± 8
Heart rate (beats/min)	92 ± 5	107 ± 27
Right ventricular end-diastolic volume (ml/m^2^)	103 ± 21	120 ± 15
Right ventricular ejection fraction	27 ± 9	30 ± 5
Pulse-pressure variation	20 ± 20	27 ± 37

Values are expressed as mean ± SD.

**Table 3:** Hemodynamic changes in patients with (responders) and without (nonresponders) increase in stroke volume after a volume challenge

	Cardiac surgery		Sepsis		*P *value		
			
	Before	After	Before	After	V	V/R	V/R/D
Stroke volume (ml/m^2^)					**0.001**		0.699
All	30 ± 9	31 ± 7	34 ± 6	36 ± 7			
Responders	24 ± 4	29 ± 4	32 ± 5	37 ± 7			
Nonresponders	32 ± 9	32 ± 8	35 ± 6	35 ± 7			
Pulmonary artery occlusion pressure (mm Hg)					**0.001**	0.280	0.701
All	13 ± 3	14 ± 4	16 ± 4	18 ± 4			
Responders	12 ± 1	14 ± 2	13 ± 4	18 ± 3			
Nonresponders	13 ± 4	15 ± 4	16 ± 4	18 ± 4			
Central venous pressure (mm Hg)					**0.001**	0.724	0.593
All	12 ± 4	13 ± 4	12 ± 5	14 ± 5			
Responders	12 ± 3	13 ± 4	10 ± 3	12 ± 3			
Nonresponders	12 ± 4	13 ± 4	13 ± 5	15 ± 5			
Mean pulmonary arterial pressure (mm Hg)					**0.001**	0.158	0.175
All	26 ± 4	28 ± 5	33 ± 7	35 ± 6			
Responders	27 ± 3	29 ± 3	32 ± 6	34 ± 7			
Nonresponders	26 ± 5	27 ± 5	34 ± 7	35 ± 6			
Mean arterial pressure (mm Hg)					**0.001**	0.206	0.255
All	67 ± 8	70 ± 9	58 ± 7	60 ± 7			
Responders	69 ± 7	74 ± 7	61 ± 7	63 ± 9			
Nonresponders	66 ± 9	69 ± 10	57 ± 7	59 ± 5			
Heart rate (beats/min)					0.071	0.438	0.343
All	92 ± 5	92 ± 4	116 ± 22	114 ± 22			
Responders	91 ± 4	91 ± 4	120 ± 24	117 ± 24			
Nonresponders	92 ± 5	92 ± 5	114 ± 22	113 ± 22			
Right ventricular end-diastolic volume (ml/m^2^)					0.245	0.385	0.378
All	103 ± 26	101 ± 25	122 ± 23	122 ± 24			
Responders	103 ± 38	101 ± 34	124 ± 20	122 ± 21			
Nonresponders	103 ± 21	101 ± 22	121 ± 25	122 ± 26			
Right ventricular ejection fraction (%)					0.108	0.055	0.836
All	30 ± 10	32 ± 10	30 ± 6	29 ± 6			
Responders	26 ± 9	28 ± 9	27 ± 5	28 ± 6			
Nonresponders	32 ± 10	33 ± 10	31 ± 6	30 ± 6			
Pulse-pressure variation (%)					0.307	0.573	0.386
All	22 ± 18	20 ± 17	34 ± 29	33 ± 28			
Responders	22 ± 17	15 ± 8	38 ± 32	38 ± 38			
Nonresponders	22 ± 19	22 ± 19	32 ± 29	30 ± 24			

Values are expressed as mean ± SD. Bold indicated significant *P *values (*P *< 0.05).

V, Effect of volume; V/R, volume-response interaction; V/R/D, volume-response-diagnosis interaction.

Nineteen (28%) of the 69 fluid challenges increased SV in 12 patients: in cardiac surgery patients, 12 of 44 fluid challenges (27%; seven patients) and in septic shock, seven of 25 fluid challenges (28%; five patients). If only fluid challenges that increased CVP by > 1 mm Hg are considered (31 of 44 fluid challenges in cardiac surgery and 22 of 25 in septic shock), SV increased in 15 (28%) of 53 fluid challenges.

### Pulse-pressure variation and volume responsiveness

**Figure 1:**
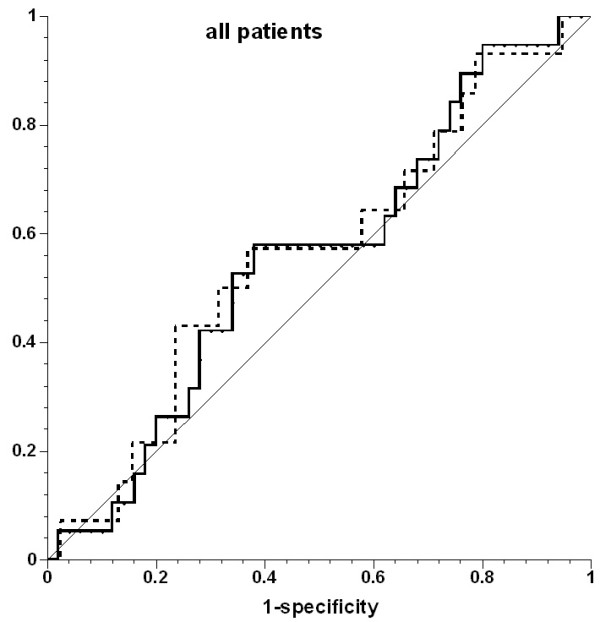
**Receiver operating characteristic (ROC) curves for prediction of ≥10% increase in stroke volume by pulse-pressure variation in all patients**. Solid line, all fluid challenges; dotted line, fluid challenges with concomitant increase in central venous pressure; thin solid line, line of identity.

**Figure 2:**
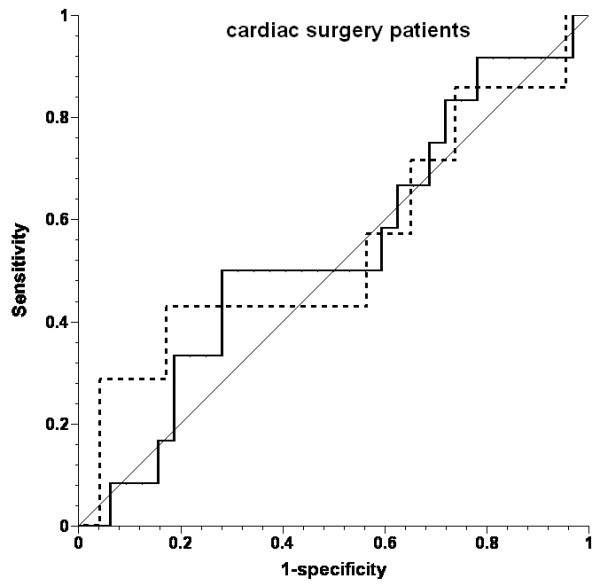
**Receiver operating characteristic (ROC) curves for prediction of ≥10% increase in stroke volume by pulse-pressure variation in cardiac surgery patients**. Solid line, all fluid challenges; dotted line, fluid challenges with concomitant increase in central venous pressure; thin solid line, line of identity.

**Figure 3:**
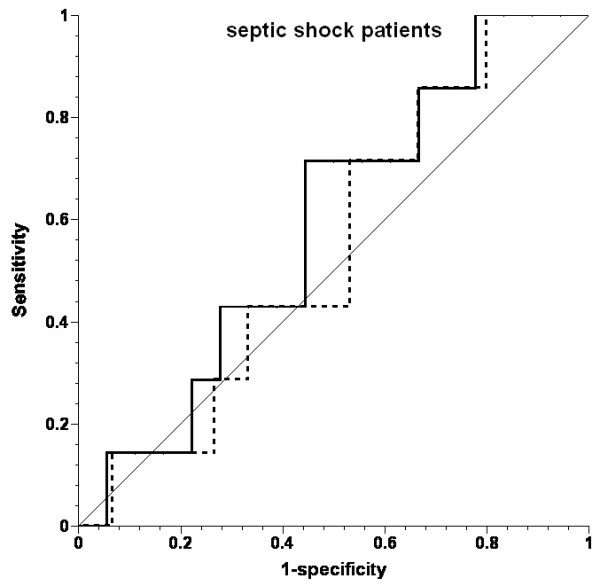
**Receiver operating characteristic (ROC) curves for prediction of ≥10% increase in stroke volume by pulse-pressure variation in septic shock patients**. Solid line, all fluid challenges; dotted line, fluid challenges with concomitant increase in central venous pressure; thin solid line, line of identity.

PPV did not predict an increase in SV. The area under the ROC curve (AUC; Figures 1,2,3) was 0.555 for the whole patient cohort, 0.539 for the cardiac surgery patients, and 0.587 for the septic shock patients (all *P *> 0.05).

Inclusion of only the first fluid challenge from each patient (n = 22) revealed 23% responders (n = 5). The area under the ROC curve for predicting a positive response by PPV was 0.447 in this case (*P *= 0.724). Inclusion of only those fluid challenges with CVP increase did not improve the prediction of increase in SV (AUC, 0.509). The threshold value for best prediction (albeit not significant) for all volume challenges was 21%.

The data were further analyzed by using a PPV threshold of ≥13%, based on other studies [22-26], and the mean threshold of 12.5% reported in a systematic review [27]. PPV was ≥13% before 46 (66%) fluid challenges: in 27 (60%) of those in cardiac surgery and in 19 (76%) in septic shock. SV increased in 13 (28%) fluid challenges: in seven (26%) in cardiac surgery and in six (32%) in septic shock. PPV was < 13% before 24 fluid challenges, and SV increased in six (25%) of them. The absolute PPV before all fluid challenges was 15 ± 13 mm Hg in cardiac surgery patients and 21 ± 18 mm Hg in sepsis patients (ns).

Right ventricular ejection fraction decreased in none of the fluid challenges resulting in increased SV, and in 44% of those in which SV did not increase (*P *= 0.0003). EDV increased in 28% of fluid challenges resulting in increased SV and in 45% of those in which SV did not increase (*P*, not significant).

## Discussion

The main finding of this study was the lack of association between PPV and volume responsiveness in patients with increased pulmonary artery pressure. This was the case both in postoperative cardiac surgery patients and in patients with septic shock, despite the very different underlying circulatory pathology. Enhanced pulse-pressure variation was present before most of the clinically indicated fluid challenges. Despite this, only about one of four fluid challenges resulted in increased stroke volume. The association was no better if only those fluid challenges were considered that were preceded by pulse-pressure variation of ≥13%, a threshold proposed by several authors, and approximately the mean value (12.5%) found in a recent systematic review [27].

Our results are in sharp contrast to those of several previous studies in septic and postoperative patients, in which pulmonary artery pressure was either not increased or not reported [6,22-26,28-33], or in which pulmonary artery pressure was markedly less increased [12,34-36]. Recently, Mahjoub *et al*. [15] reported a failure to predict fluid responsiveness by PPV in patients who had echocardiographic findings suggesting right ventricular systolic dysfunction without overt signs of right ventricular failure. We selected patients at relevant risk of acute right ventricular dysfunction due to increased pulmonary artery pressure. Our finding of reduced right ventricular ejection fraction in almost half of the nonresponders and in none of the responders supports the concept that right ventricular dysfunction contributed to the poor predictive value of PPV. Admittedly, the reliability of right ventricular ejection fraction estimation by using thermodilution catheters is controversial. Because we did not perform concomitant echocardiography, it is conceivable, conversely, that the presence and severity of right ventricular dysfunction might have been underestimated.

Failure to induce an increase in preload and failure to detect changes in stroke volume would be the most obvious alternative explanations for this controversial finding. We consider these explanations unlikely. First, any acute increase in central venous pressure in response to volume loading suggests an increase in preload and should result in an increase in stroke volume if the heart is operating in the volume-responsive part of the cardiac-function curve [20,21,37]. The increase in central venous pressure in 76% of fluid challenges strongly suggests that preload acutely increased in the majority of fluid challenges; in those without a relevant increase in central venous pressure, the fluid challenge apparently failed to increase the stressed volume, possibly due to vasodilation. Second, the percentage of responders versus nonresponders (27% versus 73%) was the same when all fluid challenges were considered. Because no clinically applicable method provides an accurate beat-to-beat measurement of cardiac output, we chose to use the continuous thermodilution method. We used the 1-minute values provided by the software, so this method does not induce a clinically relevant delay in the measured values. Hence, relevant rapid changes should not have been missed. We also eliminated all phases of spontaneous respiration by evaluating all individual tracings, and used tidal volumes large enough (9 ml/kg on average (range, 8 to 10 ml/kg)) to result in relevant intrathoracic pressure changes [36,37].

The potential of increased pulmonary artery pressure to interfere with the predictive value of pulse-pressure variation has been addressed previously, but its clinical relevance has been considered small [13,14]. This view is in sharp contrast to the frequent occurrence of high pulmonary artery pressures in the early postoperative period after cardiac surgery [16,17], as well as in septic shock [18,19]. Because the use of the pulmonary artery catheter has decreased, moderate or transient but relevant increases in pulmonary artery pressures may easily be overlooked. Our results suggest that under these conditions, PPV may erroneously suggest volume responsiveness. The recent study of Mahjoub *et al*. [15] supports this concept. Those authors considered the risk of false-positive prediction of fluid responsiveness by PPV high enough to warrant echocardiography before performing fluid challenge based on increased PPV.

The dynamic association between volume status and PPV has been advocated as an alternative to predict which patients will respond to fluid administration [12,14]. We tested this in a clinical context in which fluid infusion is unavoidable (that is, in patients with septic shock and in patients immediately after cardiac surgery). Instead of selecting patients with a specific condition believed to reflect fluid responsiveness, we chose situations in which clinicians considered volume expansion with fluids necessary. Although PPV has been well validated in the context of hypovolemia [38,39], the presence of pure hypovolemia is probably less common and more difficult to treat in patients with septic shock or after cardiac surgery, when pulmonary artery pressures are increased. In these situations, complex heart-lung interactions may be present as a consequence of either compromised myocardial function in cardiac surgery patients or both diastolic and systolic biventricular dysfunction and acute changes in pulmonary vascular resistances in patients with septic shock. Previous studies in cardiac surgery patients in whom PPV predicted volume responsiveness either have been performed right after induction of anesthesia [23,24,28-30], have included patients with normal pulmonary artery pressure [24,26,30,31], or have not reported pulmonary artery pressure [22,23,28,29,32]. In studies on sepsis patients, pulmonary artery pressure was less increased than in the present study [12,34-36] (average mean pulmonary artery pressure, 24 to 26 mm Hg before fluid challenge versus 33 mm Hg in this study) or was not reported [25,33].

We believe that our findings are real, especially considering the persisting large PPV values. These increased PPV values cannot be explained by relative hypovolemia, but are rather the consequence of an impeded right systolic function, as a consequence of intrathoracic pressure changes in a situation in which the right ventricle is already at the flat part of preload dependence. Hence, PPV had no predictive value for volume responsiveness. Furthermore, clinical use of PPV to predict volume responsiveness may misleadingly suggest volume responsiveness when increased pulmonary artery pressure compromises right ventricular function, and further volume expansion may be harmful.

We therefore performed a second, experimental study. In the accompanying article [40], we assess the effect of acutely increased pulmonary artery pressure on volume-responsiveness prediction with PPV.

A limitation of this study is the lack of airway-pressure signal recording concomitant with the hemodynamic signals. Our approach does not guarantee 100% exclusion of data from patients with spontaneous respiratory activity, although respiratory efforts with an influence on PPV should mostly be detectable in central venous and pulmonary artery pressure tracings. Recording airway-pressure signals would also have enabled us to define whether PPV was the result of an increase in PP during inspiration, a decrease during expiration, or both.

Another potential confounder is the use of a colloid rather than a crystalloid. It has been suggested that colloids may have effects on cardiac function independent of their volume-expanding effect. However, this should have tended to increase fluid responsiveness rather than to decrease it.

## Conclusions

We conclude that both early after cardiac surgery and in septic shock, patients with increased pulmonary artery pressure respond poorly to fluid administration. Under these conditions, PPV cannot be used to predict fluid responsiveness. We suggest that right ventricular dysfunction contributed to the poor response to fluids.

## Key messages

• Septic shock and post-cardiac surgery patients with increased pulmonary artery pressure respond poorly to fluid administration.

• Pulse-pressure variation does not predict fluid responsiveness in septic shock and post-cardiac surgery patients with increased pulmonary artery pressure.

• Right ventricular dysfunction may contribute to poor fluid response in such patients.

## Abbreviations

AUC: area under the curve; CVP: central venous pressure; ECG: electrocardiogram; EDV: end-diastolic volume; EF: ejection fraction; PAOP: pulmonary artery occlusion pressure; PEEP: positive end-expiratory pressure; PPV: pulse-pressure variation; ROC: receiver operating characteristic; SAPS: simplified acute physiology score; SIRS: systemic inflammatory response syndrome; SOFA: sequential organ-failure assessment; SV: stroke volume. SvO_2_: mixed venous oxygen saturation.

## Competing interests

The Department of Intensive Care Medicine has, or has had in the past, research contracts with Abbott Nutrition International, B. Braun Medical AG, CSEM SA, Edwards Lifesciences Services GmbH, Kenta Biotech Ltd, Maquet Critical Care AB, Omnicare Clinical Research AG, and Orion Corporation; and research & development/consulting contracts with Edwards Lifesciences SA and Maquet Critical Care AB. The money is/was paid into a departmental fund; no author receives/received individual fees. The past contract with Edwards Lifesciences is unrelated to and did not influence the current study.

## Authors' contributions

MWvB analyzed the data and drafted the manuscript. JT and SMJ designed and supervised the study, performed the statistics, and critically revised the manuscript. MR, FP, DT, CCG, RS, HB, and BB performed the study. All authors read and approved the final manuscript.

## Supplementary Material

Additional file 1 **Clinical indications for fluid challenges**. A table listing clinical indications for fluid challenges. Click here for file
