# Supplementary material for: Pulse-pressure variation and hemodynamic response in patients with elevated pulmonary artery pressure: a clinical study
Source: Crit Care. 2010 Jun 11;14(3):R111. doi: 10.1186/cc9060 (PMC2911757; doi:10.1186/cc9060)
Supplement: Additional file 1 — Clinical indications for fluid challenges. A table listing clinical indications for fluid challenges. [file cc9060-S1.DOC]

Additional File 1: Clinical indications for fluid challenges.

|  | Cardiac surgery (n=44)  n % | |  | Septic shock (n=25)  n % | |
| --- | --- | --- | --- | --- | --- |
| Insufficient blood pressure | 28 | 62 |  | 18 | 72 |
| Tachycardia | 0 | 0 |  | 1 | 4 |
| Peripheral vasoconstriction | 16 | 36 |  | 4 | 16 |
| Insufficient urinary output | 0 | 0 |  | 3 | 12 |
| Insufficient SvO_2_ | 3 | 7 |  | 1 | 4 |
| Insufficient CVP | 4 | 9 |  | 2 | 8 |
| Insufficient PAOP | 2 | 4 |  | 1 | 4 |
| Insufficient cardiac output | 8 | 18 |  | 1 | 4 |

SvO_2_: mixed venous oxygen saturation; CVP: central venous pressure; PAOP: pulmonary artery occlusion pressure. More than one indication per fluid challenge is possible.
